# Using Fluorescence Quenching Titration to Determine the Orientation of a Model Transmembrane Protein in Mimic Membranes

**DOI:** 10.3390/ma12030349

**Published:** 2019-01-23

**Authors:** Haihong Huang, Baosheng Ge, Shuai Zhang, Jiqiang Li, Chenghao Sun, Tongtao Yue, Fang Huang

**Affiliations:** 1 State Key Laboratory of Heavy Oil Processing, China University of Petroleum (East China), Qingdao 266580, China; 429love@163.com; 2 Center for Bioengineering and Biotechnology, College of Chemical Engineering, China University of Petroleum (East China), Qingdao 266580, China; gebaosheng@upc.edu.cn (B.G.); zhangshuaixx@gmail.com (S.Z.); ljq325@163.com (J.L.); g.sunch2010@gmail.com (C.S.)

**Keywords:** transmembrane protein, mimic membrane, insertion, orientation, fluorescence quenching titration

## Abstract

After synthesis of transmembrane proteins (TMPs), they are transferred and inserted into plasma membranes to play biological functions. Crucially, orientation of TMPs in membranes determines whether they have biological activities. In cellular environments, a number of cofactors, such as translocon, can assist TMPs to be inserted into membranes in defined orientations. During in vitro reconstitution of TMPs with mimic membranes, both insertion and orientation of TMPs are primarily determined by interactions with the membrane. Yet the knowledge is limited, hindering the in vitro applications of TMPs. Here, we take Bacteriorhodopsin (bR) as a model TMP, using fluorescence quenching titration experiment to identify orientation of bR in mimic membranes, examining effects of a number of factors, including lipid composition, pH value, ionic strength and membrane curvature. The most effective determinant is the lipid type, which modulates insertion and orientation of bR in membranes by changing the membrane surface charge and the membrane fluidity. Both the pH value and the ionic strength play secondary roles by tuning the nature of the electrostatic interaction. The membrane curvature was found to have a minor effect on orientation of bR in membranes. By comparing orientations of bR in folded and unfolded states, no obvious change was observed, informing that nascent proteins could be inserted into membranes in defined orientations before folding into the native state inside the membrane.

## 1. Introduction

The folding mechanism of water-soluble proteins has been investigated for decades and relatively well understood [1], whereas folding of membrane proteins still remains ambiguous [2,3,4,5], not only because of the lower protein concentration in membranes restraining protein extraction and purification but also due to the difficulty of reconstituting purified proteins with membranes [6]. Besides folding, inserting proteins into plasma membranes is an equally fundamental biophysical process [7]. During in vitro experiments, acquiring stable membrane-protein structures with defined orientation of proteins in membranes is of essential importance ensuring biological activities of the product. This is trivial in vivo because both folding and inserting of proteins are intelligently and efficiently assisted by a number of cofactors, such as translocon and molecular chaperone [8,9,10,11]. In the absence of cofactors, such as during in vitro recombination experiments, both insertion and orientation of proteins are expected to be mainly determined by their interactions with the membrane. However, the knowledge on how TMPs orientate in mimic membranes is still lacking.

Bacteriorhodopsin (bR) is an ideal model for studying folding and membrane interactions of transmembrane proteins (TMPs) [12,13,14,15]. It is composed of seven α helices and displays purple color in solution due to the presence of retinal that binds through a Schiff base to lysine-216 locating at the last helix. It has 248 amino acid residues with N- and C-termini pointing to opposite sides of the membrane. Previous studies have successfully captured the intermediate state in both folding and unfolding processes of bR, facilitating our understanding on membrane protein folding [16,17]. In the presence of translocon in vivo, natural bR correctly inserts into membranes, leaving the N-terminus pointing to the cell exterior. However, the question of whether bR is inserted into the membrane in folded or unfolded state still remains unclear, even controversial [2,18].

During in vitro experiments, construction of applicable mimic membranes is requisite for exploring folding, inserting and orientating of TMPs in the membrane [19,20,21]. With phospholipid as the major component, they can spontaneously assemble into different morphologies, like micelles, bilayers, vesicles and tubes. Specifically, lipid vesicle is an ideal model membrane platform for exploring insertion, especially the orientation of TMPs in the membrane, because the interior of vesicles can be easily distinguished from the exterior to identify the orientation of TMPs. This property can be well utilized by the experimental method of fluorescence quenching titration (FQT) [22]. The FQT method is based on the use of TAMRA, a fluorescent molecule with high extinction coefficient and fluorescence quantum yield [23,24], acting as a fluorescent probe and a free soluble tryptophan zwitterion as a quencher. Note that the free soluble tryptophan zwitterion cannot penetrate through the membrane, thus only quenching fluorescence of TAMRA exposed outside the vesicle. By premixing SDS to denature bR before membrane insertion, the membrane orientations of bR in folded and unfolded states can be compared, thus helping answer whether and how the folding state of bR affects its orientation in mimic membranes and implying whether bR could be inserted into membranes in folded or unfolded state.

Briefly, our experimental results demonstrate that bR can be readily inserted into mimic membranes in preferred orientations and the orientation can be modulated by varying environmental conditions. Among all considered factors, changing the lipid composition is the most capable of regulating the orientation of bR in mimic membranes. Other factors, including pH value, ionic strength and membrane curvature, were found to play secondary or minor roles in orientating bR in membranes. Experiments with denatured bR demonstrate that TMPs could be inserted into membranes in the unfolded state and complete folding inside the membrane without the assistance of cofactors in vitro.

## 2. Experimental Section

### 2.1. Vesicle Preparation

**Figure 1:**
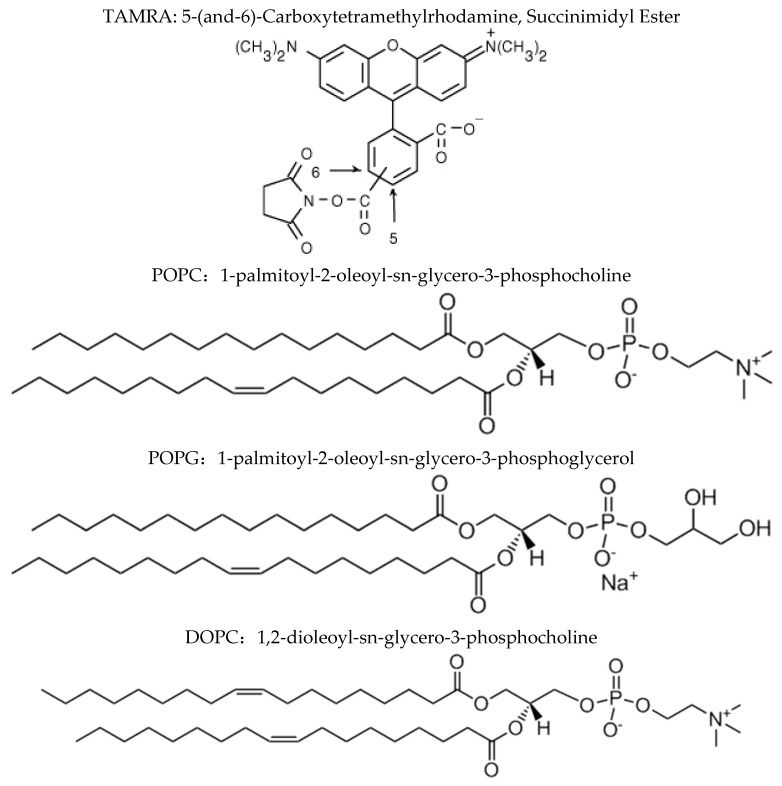
Molecular structures of both TAMRA and different types of lipids used in our experiments.

Three types of lipids, including POPC, POPG and DOPC, were purchased from Avanti Phospholipid (Avanti, AL, USA). Shown in Figure 1 is the molecular structure of each lipid type. Compared with POPC and POPG molecules, each of which contains one unsaturated tail bond, the DOPC molecule has two unsaturated bonds. Compared with POPC and DOPC molecules, both of which have no electric charge, the POPG molecule has a negative charge. We can observe the orientation of bR in mimic membranes in term of comparing their similarities and differences. The detailed process of vesicle preparation is given elsewhere and briefly described as follows [22]. First, lipids dissolved in chloroform were evaporated in vacuum for 20 min and dried overnight. Phosphate buffer was then added to obtain the multilayer lipid aggregates. Subsequently, the lipid solution was extruded 30 times through a membrane with a specified diameter to obtain lipid vesicles with a defined concentration of 6 mg/mL in phosphate buffer. 

### 2.2. DLS Measurements

The size distribution of the prepared lipid vesicles was measured by means of Dynamic Light Scattering (DLS) using a Zetasizer Nano ZS (Malvern Systems, Southborough, MA, USA). The DLS measures Brownian motion and relates it to the size of the lipid vesicles. The instrument uses a 633 nm wavelength laser beam and detects the light signal at an angle of 173°.

### 2.3. TEM Imaging

The morphology of the prepared vesicles was characterized using transmission electron microscopy (TEM) imaging, which were obtained on a JEOL-2100 UHR electron microscope (JEOL, Tokyo, Japan) at an accelerating voltage of 200 kV. In details, lipid vesicles at a concentration of 6 mg/mL were dropped on a 300-mesh copper grid. After incubation for 5 min, the excess solution was removed with filter pater. Then the grids were negatively stained with uranyl acetate (2%, w/v) aqueous solution for about 5 min. Freeze-fraction TEM was employed to analyze the morphology of lipid vesicles. A small amount of the vesicle solution was placed on a specimen holder, being frozen by quickly plunging the specimen holder into liquid ethane cooled using liquid nitrogen. Fracturing and replication were carried out on a freeze-fracture apparatus (EM BAF 060, Leica, Germany) at −140 °C. Pt/C was deposited at an angle of 45°. The replicas were transferred onto copper grid and then examined using a JEOL JEM-1400 TEM (JEOL, MA, USA) at 120 kV. More details of the method of vesicle characterization were described in our previous work [22].

### 2.4. Preparation of bR-TAMRA and Reconstitution into Vesicles

bR was first purified from *Halobacterium salinarum* using the method developed by Oesterhelt et al. [25] The purified bR with a concentration of 1 mg/mL was then labeled with TAMRA on its N-terminus. We note that a number of lysines exist in the protein sequence, which provides amino groups for TAMRA labeling. However, it has been known that different types of amines can be selectively labeled by controlling the reaction conditions. For example, by decreasing the pH value to 6.4, the succinimidyl ester group could react preferentially with the N-terminal amines rather than the primary amines [26]. In detail, the buffer was exchanged into the 100 mM phosphate buffer with a pH value of 6.4 and a salt (NaCl) concentration of 100 mM. Then TAMRA dissolved in buffer was added into the protein solution, which was incubated for 1h with stirring and covered with aluminum foil. TAMRA was removed by size exclusion chromatography immediately after being quenched with 1 M Tris buffer (Tris-HCl and Tris base). After that, the TAMRA labeled bR was further characterized using uv-vis and fluorescence spectrophotometry and the labeling sites of bR were identified using tryptic-digested mass spectrum analysis [27].

The bR labeled with TAMRA was then added into the vesicle solution to get recombination with membranes. To acquire the proportion of labeled bR being inserted into mimic membranes, we detected the fluorescence anisotropy value on a FluoroMax-4 fluorescence spectrometer (Horiba Jobin Yvon, (Horiba Jobin Yvon, Kyoto, Japan). The excitation and emission wavelengths were set to 540 nm and 576 nm, the slits of which were 4 nm and 3 nm, respectively. In fluorescence anisotropy experiments, the vertically and horizontally polarized emission intensities were background-corrected with a blank containing the lipid vesicle suspension as a reference.

We note that earlier experiments reported that detergents were applied in the reconstitution of TMPs with lipid vesicles. However, our present experiments suggest that detergents are not required for reconstitution of bR with vesicles. Instead, bR purified from *halobacterium* could be reconstituted into different lipid bilayers through a very simple and efficient way. We used the sucrose density gradient centrifugation to determine the efficiency of membrane protein reconstitution (Figure S1). It was found that all the bR proteins were successfully reconstituted into vesicles by direct mixing. Here, the direct reconstitution without detergent may be due to the existence of residual amount of lipids from purple membrane, where bR was purified from.

In the reconstitution of unfolded bR with vesicles, bR was first unfolded with 0.3% SDS. The unfolded bR was then mixed with vesicle solution with volume ratio of 1:10, corresponding to a final SDS concentration of 0.03%, where bR can refold. According to our experiments, at this SDS concentration, the vesicles are stable (Figure S2).

### 2.5. Fluorescence Quenching Titration Experiments

FQT experiment was carried out by measuring the fluorescence intensity of the TAMRA-bR mixed with an increasing concentration of tryptophan in phosphate buffer. The excitation wavelength was 535 nm and emission spectra were detected between 540 and 700 nm. The fluorescence intensity at 580 nm was analyzed by fitting the data with the Stern-Volmer equation,

(1) $$\frac{I_{0}}{I}=1+K_{q}[Q]$$

where *I*_0_ and *I* are the fluorescence intensities of bR-TAMRA in the absence and presence of the quencher, respectively. [*Q*] is the quencher concentration and *K_q_* is the Stern-Volmer quenching constant.

Assuming that the percentage of the TAMRA-bR with N-terminus pointing inside the vesicles is *x*, the value of *x* can be determined by the following method, as we reported previously [22]. First, we recombined TAMRA-bR with lipid vesicles, after which an increasing concentration of tryptophan was added into the sample. We detected the fluorescence intensity under each concentration to establish the relation between *I* and [*Q*]. According to Equation (1), the relationship between [*Q*] and fluorescence intensity follows the equation,

(2) $$\frac{I_{0}}{I}=\frac{1+K_{q}[Q]}{(1+K_{q}[Q])x+1-x}$$

Given the previously determined value of *K_q_*, the value of *x*, namely, the percentage of the TAMRA-bR with N-terminus pointing inside the vesicles can be calculated by fitting the quenching experimental data.

## 3. Results and Discussion

### 3.1. Labeling of bR with TAMRA and Reconstitution into Vesicles

**Figure 2:**
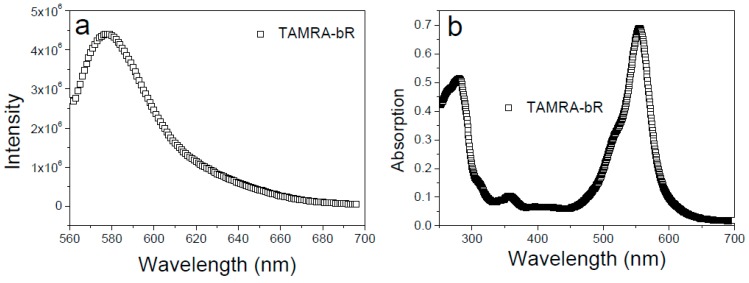
Fluorescence spectra (**a**) and UV-Vis absorption spectra (**b**) of bR proving its successful labeling with TAMRA.

**Figure 3:**
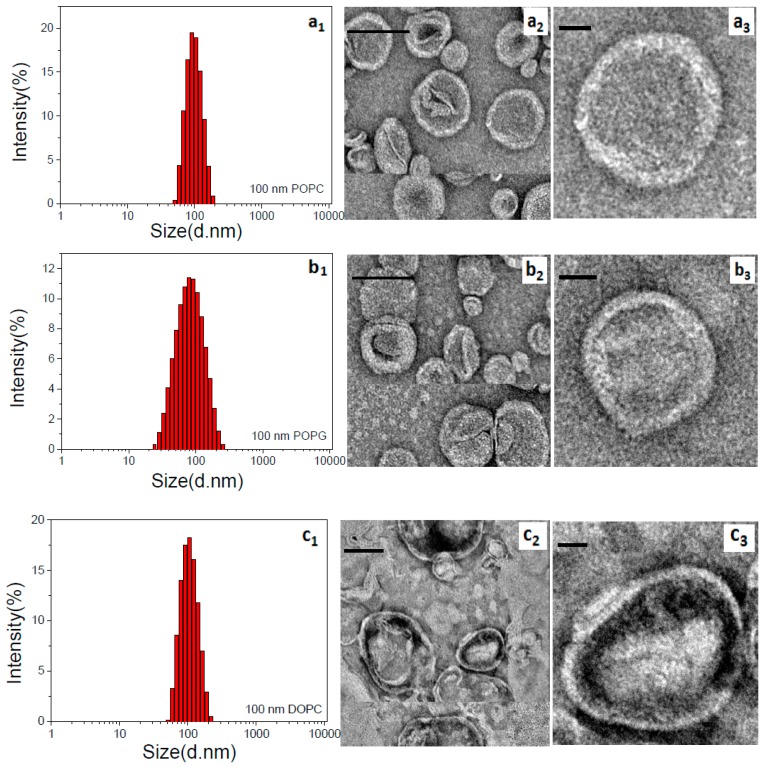
DLS data and Negative-Stain TEM images of lipid vesicles prepared by extrusion (**a**) 100 nm POPC; (**b**) 100 nm POPG; (**c**) 100 nm DOPC.

For determining the orientation of bR in mimic membranes using the FQT method, the purified bR was labeled with TAMRA only on its N-terminus. We note that a number of lysines exist in the protein sequence, which provides primary amino groups for TAMRA labeling. To probe whether bR has been successfully labeled with TAMRA, we detected the fluorescence spectra and UV-Vis absorption spectra of the product. As shown in Figure 2, the fluorescence emission peak at 576 nm and the characteristic absorption peak at 560 nm both prove that bR has been successfully labeled with TAMRA. To exclude the unexpected fluorescence labeling on lysines, the pH value of reaction buffer was strictly set as 6.4 [26] and the labelling efficiency was kept low. After fluorescence labeling, tryptic-digested mass spectrometry analysis was performed and no TAMRA-labelled intramolecular lysine was observed (Figure S3). Both DLS and TEM results suggested that three types of lipid vesicles with the uniform particle size distribution have been prepared at room temperature (Figure 3). Specifically, the average particle size of vesicles is about 100 nm and the intact morphology of vesicles was clearly visualized from the TEM images. 

**Figure 4:**
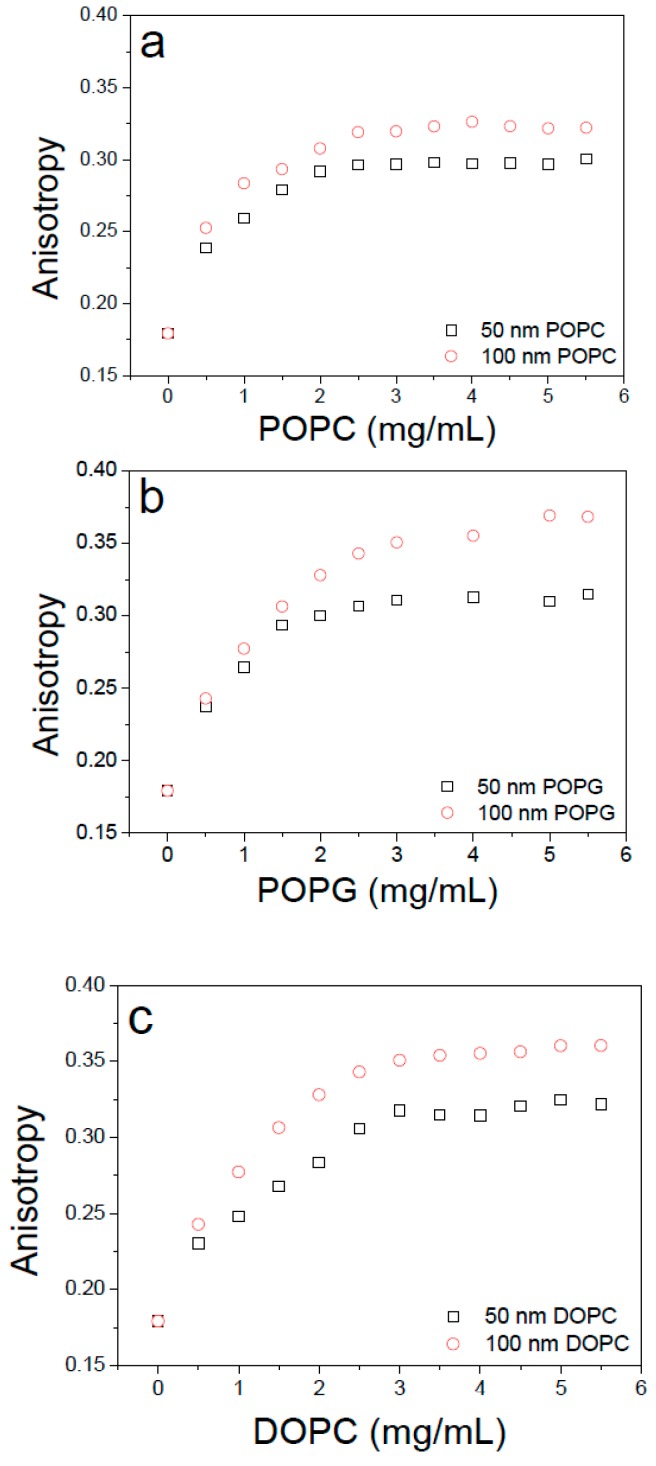
The fluorescence anisotropy of TAMRA-bR incorporated in an increasing concentration of lipid vesicles. Three types of lipids were used, including POPC (**a**); POPG (**b**) and DOPC (**c**), the 50 nm and 100 nm indicate the size of mimic membrane. We have conducted a systematic study of the concentration of phospholipids impact on the scattered light of mimic membranes in our early studies and obtained the optimal concentration of bR to be 4 µM and the mimic membranes to be 3 mg/mL.

After that, we measured the fluorescence anisotropy of the TAMRA-bR incorporated with lipid vesicles of increasing concentration to obtain the molar ratio of complete reconstitution. As shown in Figure 4, the value of fluorescence anisotropy was below 0.2 without lipid vesicles, considering the influence of existing surfactants. As we gradually increased the concentration of lipid vesicles, more TAMRA-bRs were incorporated into the vesicles, thus increasing the value of anisotropy, until all TARMA-bRs were incorporated into the vesicles when the ratio between proteins and lipids was larger than 4:3. We thus obtained the optimal concentration of bR to be 4 μM and that of mimic membranes to be 3 mg/mL.

### 3.2. Lipid Composition Modulates Orientation of bR in Mimic Membranes

Before conducting the FQT experiments, three possible orientations of bR in mimic membranes were presumed to exist. As described above, our FQT experiments were based on the fact that the fluorescence of TAMRA can be efficiently quenched by free soluble tryptophan zwitterion, which could not penetrate through the membrane to quench fluorescence of the TAMRA being inserted into vesicles. Since only the N-terminus of bR was labeled with TAMRA, theoretically, the final orientation of bR being inserted into mimic membranes can be identified by the fluorescence intensity after adding the quencher. If all C termini are inserted into the vesicle, leaving N termini exposed outside the vesicle, the fluorescence intensity should be effectively reduced upon increasing the free tryptophan concentration. Conversely, the fluorescence intensity will be nearly unchanged if all N termini are inserted into the vesicle. More generally, the fluorescence intensity could be partially reduced by the quencher, suggesting that a proportion of N termini are inserted into vesicles.

**Figure 5:**
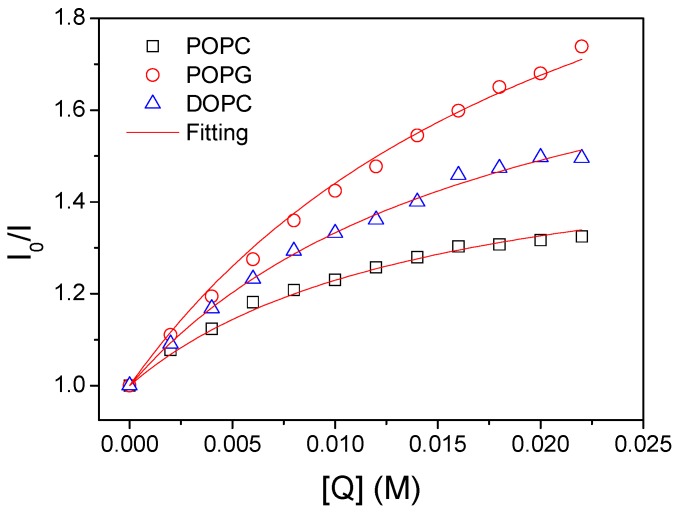
The fluorescence quenching titration data for TAMRA-bR incorporated into three types of lipid vesicles.

According to Equation (2) (see the Experimental section), calculating the orientation distribution of bR requires the value of *K_q_*. We thus first performed FQT experiments in the absence of lipid vesicles. The linear ratio between *I*_0_/*I* and [*Q*] is given in Figure S4. By fitting the quenching data, the quenching constant (*K_q_*) was determined to be 1.0 × 10^7^ M^−1^. After that, we reconstituted TAMRA-bR into POPC, POPG and DOPC vesicles. The detected values of *I*_0_/*I* as a function of [*Q*] are given in Figure 5. By fitting the quenching data using Equation (2), we obtained the value of *x* to be 0.64, 0.41 and 0.52 respectively. Note that single experiment cannot rigorously suggest the preferential orientation of bR in lipid vesicles. We thus repeated multiple titration experiments to determine the average value of *x*. Importantly, similar results were obtained by repeating multiple experiments, with the error being ignored to suggest that bR preferentially inserts its N terminus into the POPC membrane (*x* = 0.63 ± 0.02), whereas more C termini were inserted into membranes composed of POPG molecules (*x* = 0.40 ± 0.01). The value of 0.53 ± 0.01 indicates that bR has no preferential orientation in DOPC membranes. 

**Figure 6:**
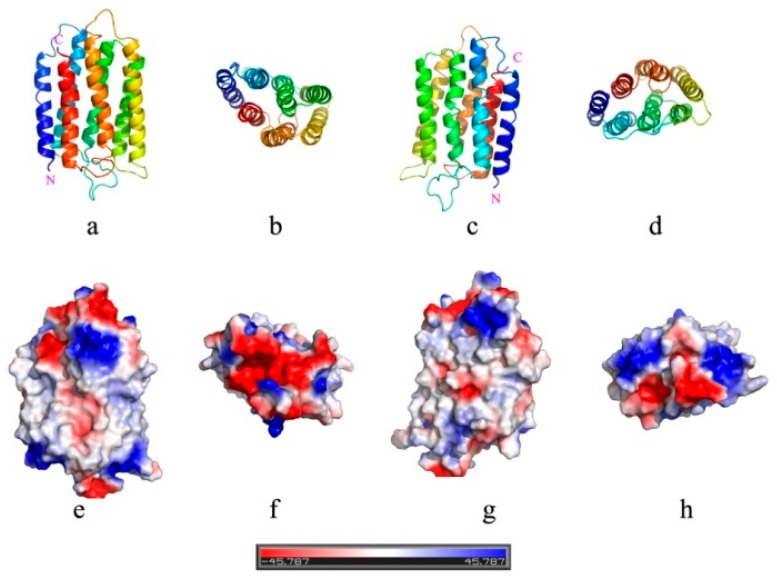
Structural and electrostatic properties of bR (PDB code: 1AP9). (**a**–**d**) Ribbon representations of the crystal structure from different views. (**e**–**h**) Electrostatic surface potential calculated with the aid of the Protein Continuum Electrostatics tool. b and f are the top views with the C terminus pointing upwards, whereas d and h are those viewing form the N terminus.

Essentially, why simply changing the lipid component could generate such distinct orientation of bR in the membrane? In the absence of translocon, both insertion and orientation of bR should be solely determined by their interactions with the membrane. Our previous molecular dynamics simulations on insertion of transmembrane peptides into membranes suggested that the first stage of membrane attachment is driven by the electrostatic interactions, whereas the subsequent insertion is synergistically or competitively affected by both the electrostatic interaction and the hydrophobic interaction [22]. In the current system, the preferential orientation of bR in mimic membranes can be analyzed based on the same consideration. By comparing the molecular structures of POPC and POPG having the same tail group (Figure 1), the membrane surface charge apparently plays a dominant role in regulating the insertion and orientation of bR in the membrane. Specifically, each POPG molecule has one negative charge, while POPC is electroneutral. Shown in Figure 6 is the electrostatic surface potential of bR being calculated with the aid of the Protein Continuum Electrostatics tool [28]. It appears that the region near the C terminus of bR has many positive charges, whereas the side of N terminus is nearly electroneutral. It is thus expected that the positively charged C termini of bR should be preferentially attracted toward the POPG membrane, accordingly having a higher probability to be inserted into the membrane driven by hydrophobic interactions between nonpolar residues of bR and lipid tails. Such explanation is consistent with our previous simulations and fits our experimental finding that more C termini of bR point inside the POPG vesicles (*x* = 0.40 ± 0.01). In vivo conditions, natural bR can correctly insert its C-terminus into membranes, leaving the N-terminus pointing to the cell exterior in vivo [29,30]. This process should be facilitated by some translocon or molecular chaperones.

However, above analysis does not interpret why more N termini of bR were inserted into the POPC vesicles, since there are nearly no electrostatic interactions between each terminus of bR and the POPC membrane. We assumed that increasing contact of C termini with the neutrally charged POPC membrane could induce strong electrostatic repulsion between C termini of adjacent bR proteins, thus hindering their contact and insertion into the membrane. By contrast, the N terminus of bR has nearly no net charge (Figure 6h), thus having a larger proportion to contact with and insert into the POPC membrane.

There are some properties of the composition of lipid, such as the charge of the lipid, the length of hydrophobic alkyl chain and unsaturation which decide the assembly potential, thickness and curvature of mimic membranes. What’s more, these properties of lipid have an impact on orientation, folding, structure and function of membrane proteins in the mimic membrane. Compared with POPC and POPG molecules (16:0, 18:1), the length of two hydrophobic alkyl chain of the DOPC molecule are 18. Membrane protein will insert into membrane bilayers at high inclination angles with the decrease in thickness of membrane, to make up the insufficient of thickness of hydrophobic environment. Besides, compared with POPC and POPG molecules, each of which contains one unsaturated tail bond, the DOPC molecule has two unsaturated bonds, thus making it more flexible and the membrane more fluidic and permeable than the other two types of lipid vesicles. It has been convincingly proved by experiments that higher fluidity and permeability could more easily generate lipid defects on membranes [31], thus permitting bR insert both termini into the membrane. Our experimental data support this analysis that no apparent selectivity of orientation of bR in DOPC vesicles was observed. Besides, the inherent structural asymmetry between two termini of bR should have minor effect on its orientation in the membrane.

### 3.3. Effects of Other Environmental Factors

Besides the lipid composition that can be used to regulate the orientation of bR in mimic membranes, other factors, such as ionic strength, pH value and membrane curvature, were expected to influence the property of bR-membrane interactions, thus modulating both the insertion and orientation of bR in lipid vesicles. 

Having analyzed that the final orientation of bR in membranes is determined by the electrostatic interactions, not only between the positively charged C terminus of bR and negatively charged lipid headgroups but also between C termini of different bRs, it can be expected that the ionic strength could modulate the orientation by influencing the electrostatic interactions. Here, for both POPC and POPG membranes, three ionic strengths (10 mM, 50 mM and 250 mM) were used to compare the orientation of bR in membranes. Since POPC molecule has no net charge on its headgroup, increasing ionic strength from 10 mM to 50 mM and 250 mM was found to have insignificant effect on the orientation, considering the measuring error (Table 1, for details see Figure S5). For POPG vesicles, it was found that increasing the ionic strength could decrease the preference of C terminus of bR being inserted into vesicles. This was expected because the preference of C terminus pointing inside the POPG vesicle is ascribed to the coulomb attraction between the positively charged C terminus and the negatively charged POPG headgroup (Figure 5). By increasing the ionic strength, this attraction is reduced, thus decreasing the preference of orientation of bR in POPG membranes (*x* = 0.48 ± 0.01). This observation further manifests the importance of electrostatic interactions between TMPs and membranes in determining both insertion and orientation of TMPs in mimic membranes. 

Similar results were observed for the effect of the pH value, being another environmental factor that potentially influences interactions between bR and lipid membranes. We have shown that, under pH value of about 7.0, POPC vesicles preferentially permit bR to insert its N terminus into the vesicles (*x* = 0.63 ± 0.02), while the POPG membrane attracts positively charged C terminus of bR to be inserted into the vesicles (*x* = 0.40 ± 0.01). Similar with the effect of the ionic strength, the electrostatic interactions between bR and the membrane can be shielded under both acidic and alkaline conditions. As expected, we observed that the preference of orientation of bR in membranes for both lipid types became less obvious (Table 2, for details see Figure S6).

During in vitro experiments of membrane-protein interactions, membrane curvature is expected to be an important factor influencing the interactions by changing the bending stress or surface tension of membranes. Theoretically, smaller vesicles having larger curvatures are under larger bending stress that facilitates insertion of membrane proteins. Our experiments also demonstrated that under the same concentration of lipid vesicles, more bR proteins were found to be inserted into vesicles with smaller size. However, by using vesicles of two diameters (50 nm and 100 nm), no obvious change of orientation of bR in membranes was observed (Table 3, for details see Figure S7). Nevertheless, elucidating effect of the membrane curvature on orientation of TMPs requires more experimental data using vesicles with wider size distribution and other types of TMPs and this will be pursued in our future work.

### 3.4. Folding States of bR Being Inserted into Vesicles

To date, an important question of whether TMPs insert themselves into the membrane in folded or unfolded state is still not well understood. In one pathway, the nascent peptide chains first fold being assisted by the molecular chaperone [32], then insert themselves into membranes in the folded state with the help of translocon [8]. In another pathway, the nascent proteins are first inserted into the membrane in the unfolded state before folding facilitated via interactions with surrounding lipids [18]. Discriminating these two mechanisms requires systematic investigations on interactions between membranes and proteins in different folding states. Here, our established method determining orientation of bR in mimic membranes may provide a clue to help answer this question by comparing orientations of bR proteins in folded and unfolded states. Before recombination with the POPC vesicles, 0.3% of SDS was added to denature bR. The denatured bR was then mixed with POPC vesicles and the SDS was diluted down to 0.03%, where bR can refold. In our previous work, it was shown that the folding of bR took 2 h, while this work shows that the insertion of bR into the vesicles need only 9 min. This demonstrate that unfolded bR can insert into the vesicles and refold wherein (Figure S8). By comparing with that in the absence of SDS, interestingly, no obvious change of orientation was observed (Figure S9), indicating that bR could insert its N-terminus into the POPC membrane in both folded and unfolded states. Note that there exists evidence suggesting that the SDS-denatured bR probably still contains four transmembrane helices and the covalent bond between retinal and lysine cannot be effectively broken by SDS [33,34,35]. Therefore, SDS could only partially induce the conformational change of bR, which may refold under diluted concentration of SDS. Nevertheless, our results point to an important indication that bR proteins could be inserted into membranes in the fully or partially unfolded state and complete folding inside the membrane.

### 3.5. Caveats and Drawbacks

It should be noted that although our FQT experiments provide insight into determining orientation of TMPs in mimic membranes, it is worth putting a caveat on these results. First, the optimal concentrations of bR and mimic membranes were determined ensuring saturate incorporation of bR into lipid vesicles (Figure 4). However, we cannot strictly exclude the possibility that more or less bR molecules are not incorporated into or dissociated from the membrane, due to thermal fluctuations, thus being exposed outside the vesicle to be easily quenched by tryptophan. This effect should be insignificant because incorporation of TMPs into membranes is always driven by favorable interactions. Second, our analysis of the FQT data is based on the hypothesis that TMPs including bR incorporated into lipid vesicles adopt two possible orientations inside the membrane, that is, either C or N terminus of bR is inserted into vesicles, leaving the other terminus exposed outside the vesicle. However, there may exist another case that bR locates in parallel with the membrane plane to expose both termini outside the vesicle. This case is rare because the transmembrane state of bR is much more energetically favorable than other orientations. Third, even if only the C terminus of bR is inserted into membranes, the N-terminal TAMRA might be occasionally immersed into head-groups of lipids to avoid quenching by tryptophan. Our joint experimental and simulation study revealed that both termini of TMPs inserted into membranes are frequently exposed to get contact with molecules outside the membrane [20]. Also since SDS is a detergent, in this study SDS was used as defolding agent, which would be expected to influence the lipid properties. According to our previous study, there is not observable change in the size of lipid vesicles in the presence of SDS up to 0.3% (see Figure S2), which suggests that the lipid vesicles are stable with the addition of SDS. Under the working conditions in this work, the concentration of SDS is 0.03%, which is not expected to destroy the vesicles. All these cases, despite rare or having minor effects, should be considered comprehensively with caution before determining orientation of any TMP in mimic membranes.

## 4. Conclusions

In summary, we have used fluorescence quenching titration experiment to determine the orientation of a model transmembrane protein (TMP), namely bR, in mimic membranes. By using different lipid components, our results suggest that electrostatic interactions between charged residues of TMPs and lipid headgroups critically determine which terminus is first captured by the membrane, thereby having a higher probability to be inserted into the membrane, though further insertion could be influenced by other factors, such as hydrophobic interactions between nonpolar residues and lipid tails [22]. It is found that such critical electrostatic interactions can be somewhat shielded by increasing the ionic strength or changing the pH value of solvent. By changing the vesicle size as a modulator of membrane curvature, no obvious change of orientation was observed. Finally, we compared orientations of bR in mimic membranes with and without SDS, finding that bR can be inserted into membranes in both folded and unfolded states. This observation concurs with other findings to support the idea that similar TMPs could be inserted into membranes in the unfolded state and subsequently fold into the native state inside the membrane. Our results in line with previous research that natural bR correctly inserts its C-terminus into membranes, leaving the N-terminus pointing to the cell exterior in vivo. Inspired by this research, future research on modulating orientations of other TMPs in membranes is required to further understand what internal and external factors cooperatively or competitively impact orientation of TMPs in membranes. These results would promote wide in vitro applications of TMPs in different areas of science and technology, such as biosensor and drug delivery.

## Figures and Tables

**Table 1:** The calculated orientation distribution of bR in POPC and POPG vesicles under different ionic strengths. (The value of *x* indicates the percentage of the TAMRA-bR with N-terminus pointing inside the vesicles).

Ionic Strength	*x*
POPC 10 mM	0.64 ± 0.01
POPC 50 mM	0.66 ± 0.03
POPG 10 mM	0.41 ± 0.01
POPG 50 mM	0.49 ± 0.01

**Table 2:** The orientation distribution of bR in POPC and POPG vesicles under different pH values. (the value of *x* indicates the percentage of the TAMRA-bR with N-terminus pointing inside the vesicles).

pH	*x*
POPC pH 6.2	0.64 ± 0.03
POPC pH 8.0	0.57 ± 0.02
POPG pH 6.2	0.48 ± 0.01
POPG pH 8.0	0.44 ± 0.01

**Table 3:** The orientation distribution of bR in three types of lipid vesicles with different curvatures. (the value of *x* indicates the percentage of the TAMRA-bR with N-terminus pointing inside the vesicles).

Lipid Vesicle Size	*x*
POPC 50 nm	0.64 ± 0.01
POPC 100 nm	0.64 ± 0.01
POPG 50 nm	0.43 ± 0.01
POPG 100 nm	0.40 ± 0.01
DOPC 50 nm	0.57 ± 0.01
DOPC 100 nm	0.53 ± 0.01

## Supplementary Materials

The following are available online at http://www.mdpi.com/1996-1944/12/3/349/s1, Figure S1: Reconstitution of bR revealed by sucrose density gradient centrifugation. (A) bR from Halobacterium salinarium; (B) The constitution method by mixing bR with POPC vesicles, Figure S2: Size distribution of POPC vesicles (100 nm) at different SDS concentration, Figure S3: Enzyme-digested mass spectrometry analysis of bR after TAMRA labeling. The experiments were performed as described in the supplementary methods at the National Facility for Protein Science of Shanghai. The protein was digested with Trypsin (R/K cleavage specificity) and Chymotrypsin, respectively. The latter is a serine endoproteinase that specifically cleaves peptide bonds at the C-termini of Tyr, Phe, Trp and Leu. Met, Ala, Asp and Glu may be cleaved at a much lower rate. All the digested peptides were then analyzed with Mass spectrum. The identified amino acids are highlighted in grey and the identified peptides are listed as the blue lines underneath. The experiments were carried out for three times and the results show that more than 98% of the intramolecular lysines were covered and recognized and no intramolecular lysine labeling was detected. Combining with Figure 2, it can be deduced that only N terminal amino groups were labeled with TAMRA, Figure S4: The fluorescence quenching titration data of bR in the absence of lipid vesicles, Figure S5: Fluorescence quenching titration data for bRs in both POPC (a) and POPG (b) vesicles under three different ionic strengths, Figure S6: Fluorescence quenching titration data for bRs in both POPC (a) and POPG (b) vesicles under pH values, Figure S7: Fluorescence quenching titration data for bRs in POPC (a), POPG (b) and DOPC (c) vesicles with different curvatures, Figure S8: CD spectroscopy of bR proteins with and without SDS, suggesting that bR can partially denatured by SDS, Figure S9: Fluorescence quenching titration data for bRs in POPC vesicles with and without SDS.
