# Supplementary material for: Using Fluorescence Quenching Titration to Determine the Orientation of a Model Transmembrane Protein in Mimic Membranes
Source: Materials (Basel). 2019 Jan 23;12(3):349. doi: 10.3390/ma12030349 (PMC6384929; doi:10.3390/ma12030349)
Supplement: Supplementary file 1 [file materials-12-00349-s001.pdf]

materials

Supplementary information

Using Fluorescence Quenching Titration to Determine the Orientation of a Model Transmembrane Protein in Mimic Membranes

Haihong Huang [1,2,†], Baosheng Ge [2,†], Shuai Zhang [2], Jiqiang Li [2], Chenghao Sun [2], Tongtao Yue [1,2,*] and Fang Huang [1,2,*]

1 State Key Laboratory of Heavy Oil Processing, China University of Petroleum (East China), Qingdao 266580, China; 429love@163.com
2 Center for Bioengineering and Biotechnology, College of Chemical Engineering, China University of Petroleum (East China), Qingdao 266580, China; gebaosheng@upc.edu.cn (B.G.); zhangshuaixx@gmail.com (S.Z.); ljq325@163.com (J.L.); g.sunch2010@gmail.com (C.S.)
† These authors contributed equally.
* Correspondence: yuett@upc.edu.cn (T.Y.); fhuang@upc.edu.cn (F.H)

1. Enzyme-Digested Mass Spectrometry Analysis

The enzyme-digested mass spectrometry analysis of bR after TAMRA labeling were performed at the National Facility for Protein Science of Shanghai. Protein precipitation and digestion: bR proteins were precipitated with trichloroacetic acid (TCA). The protein pellet was dried by using a Speedvac for 1–2 min. The pellet was subsequently dissolved in 8 M urea, 100 mM Tris-HCl, pH 8.5. TCEP (final concentration is 5 mM) (Thermo Scientific) and Iodoacetamide (final concentration is 10 mM) (Sigma) for reduction and alkylation were added to the solution and incubated at room temperature for 20 and 15 min, respectively. The protein mixture was diluted four times and digested with Trypsin or Chemotrypsin at 1:50 (w/w) (Promega, http://www.promega.com/). LC/tandemMS (MS/MS) analysis of peptide: The peptide mixture was analyzed by a home-made 25 cm-long pulled-tip analytical column (75 μm i.d.) which packed with 3 μm reverse phase beads (AquaC18, Phenomenex, Torrance, CA, USA), the column was then placed in-line with an Easy-nLC 1200 nano HPLC (Thermo Scientific, San Jose, CA, USA) for mass spectrometry analysis. Mass Spectrometry Condition: data-dependent tandem mass spectrometry (MS/MS) analysis was performed with a Q Exactive Orbitrap mass spectrometer (Thermo Scientific, San Jose, CA, USA). Peptides eluted from the LC column were directly electrosprayed into the mass spectrometer with the application of a distal 2-kV spray voltage. A cycle of one full-scan MS spectrum (m/z 300–1800) was acquired followed by top 20 MS/MS events, sequentially generated on the first to the twentith most intense ions selected from the full MS spectrum at a 27% normalized collision energy. The number of microscans was one for both MS and MS/MS scans and the maximum ion injection time was 50 and 100 ms, respectively. The dynamic exclusion settings used were as follows: charge exclusion, 1 and >8; exclude isotopes, on; and exclusion duration, 30 s. MS scan functions and LC solvent gradients were controlled by the Xcalibur data system (Thermo Scientific). Data Analysis: The acquired MS/MS data were analyzed against an UniProtKB drosophila database (database released on Nov.11, 2016) using Peaks studio (Peaks, http://www.bioinfor.com/peaks-studio/). Peptide mass values and sequence information from the LC—MS/MS experiments were used in the MS/MS ion search taking into account the Carbamidomethyl-Cys as fixed modification (+57.0215). Met oxidized (+15.9949) and TAMRA-Lys (with N-term TAMRA-X, +412.44) as variable modifications. The maximum missed cleavage of 1, and a precursor ion and fragment ion mass tolerance of ±20 ppm and 0.5 Da, respectively.

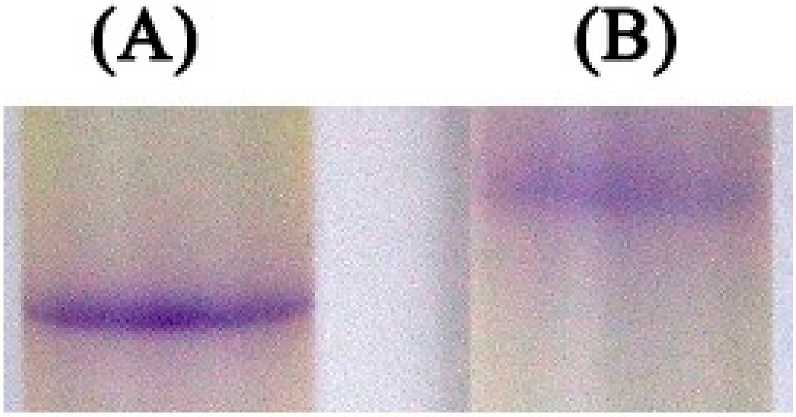

Figure S1. Picture of of bR bands separated in sucrose density gradient. (**A**) bR from Halobacterium salinarium; (**B**) The constitution method by mixing bR with POPC vesicles.

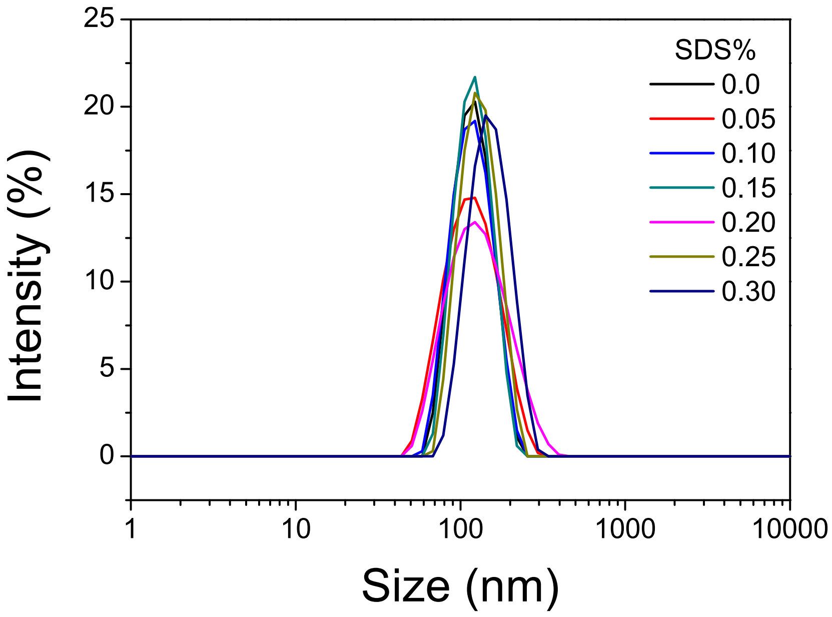

Figure 2. Size distribution of POPC vesicles (100 nm) at different SDS concentrations.

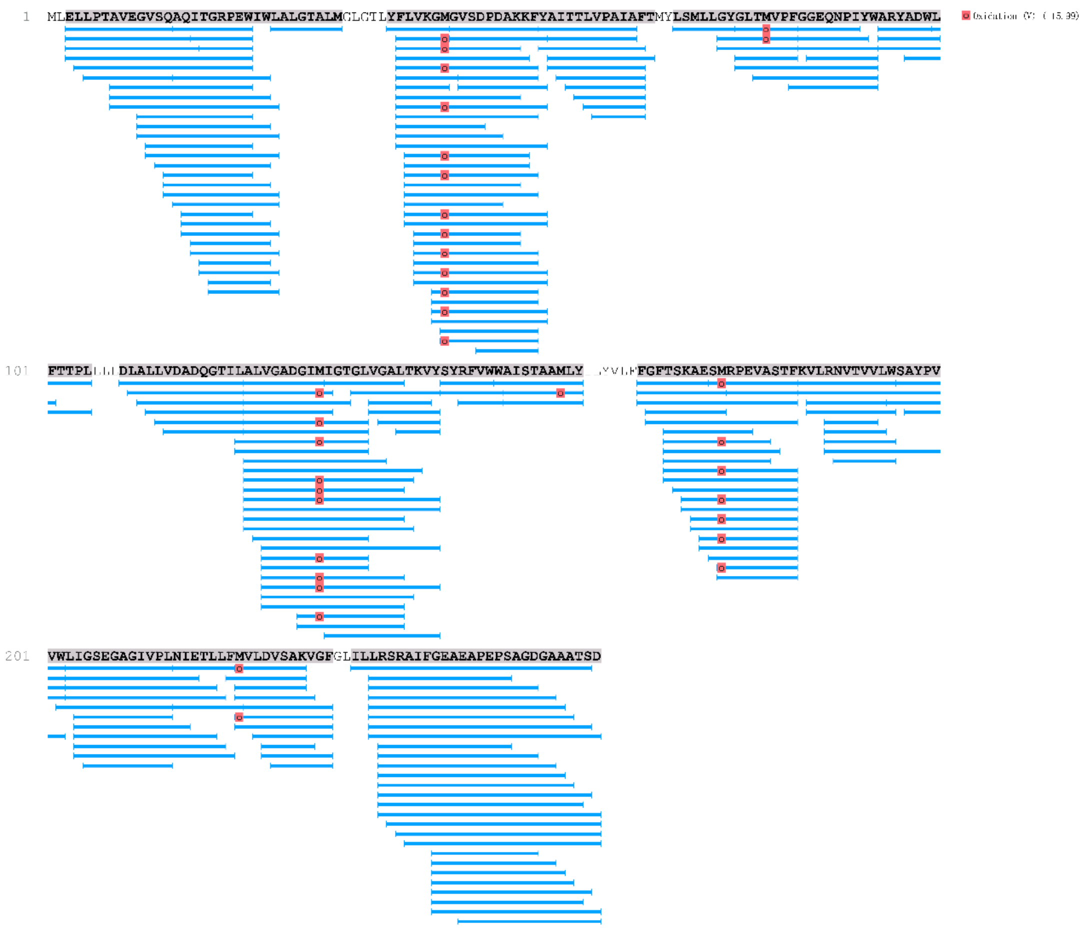

Figure S3. Enzyme-digested mass spectrometry analysis of bR after TAMRA labeling. The experiments were performed as described in the supplymentary methods at the National Facility for Protein Science of Shanghai. The protein was digested with Trypsin (R/K cleavage specificity) and Chymotrypsin, respectively. The latter is a serine endoproteinase that specifically cleaves peptide bonds at the C-termini of Tyr, Phe, Trp and Leu. Met, Ala, Asp and Glu may be cleaved at a much lower rate. All the digested peptides were then analyzed with Mass spectrum. The identified amino acids are highlighted in grey, and the identified peptides are listed as the blue lines underneath. The experiments were carried out for three times, and the results show that more than 98% of the intramolecular lysines were covered and recognized, and no intramolecular lysine labeling was detected. Combining with Figure 2, it can be deduced that only N terminal amino groups were labeled with TAMRA.

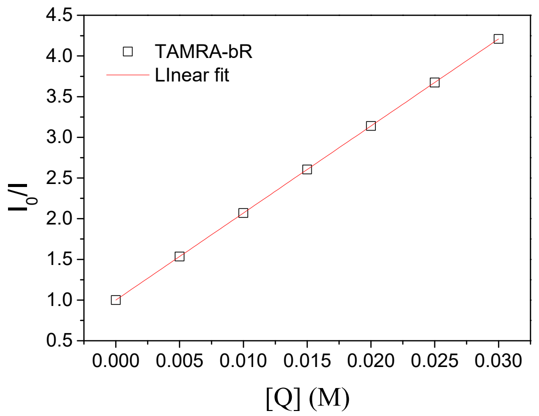

Figure S4. Fluorescence quenching titration data for bR in the absence of lipid vesicles.

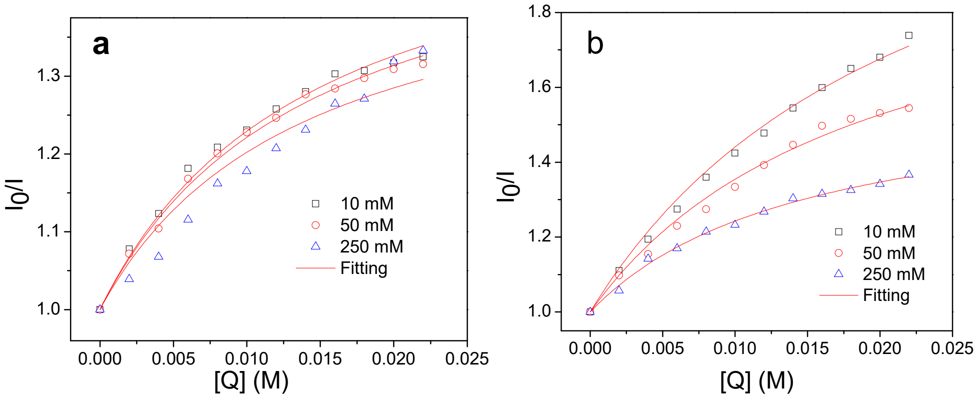

Figure S5. Fluorescence quenching titration data for bR in both POPC (**a**) and POPG (**b**) vesicles under three different ionic strengths.

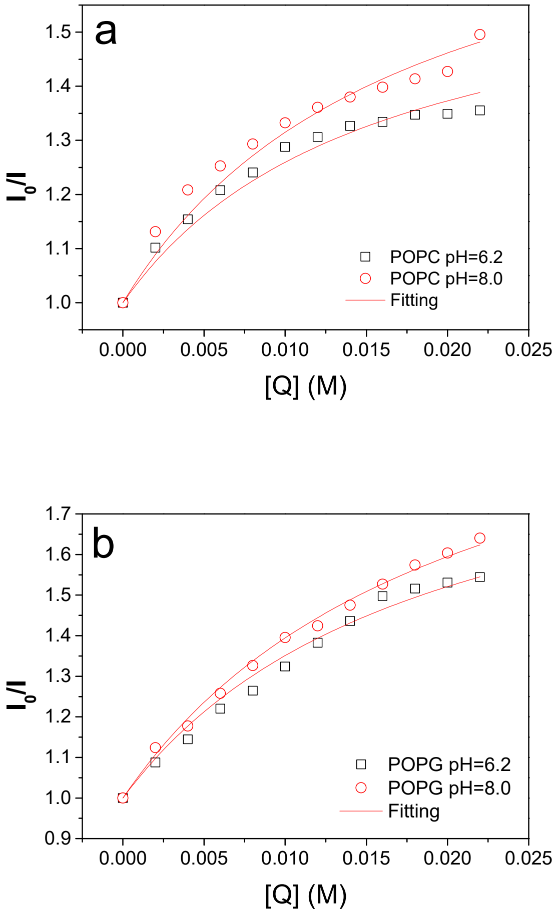

Figure S6. Fluorescence quenching titration data for bR in both POPC (**a**) and POPG (**b**) vesicles under pH values.

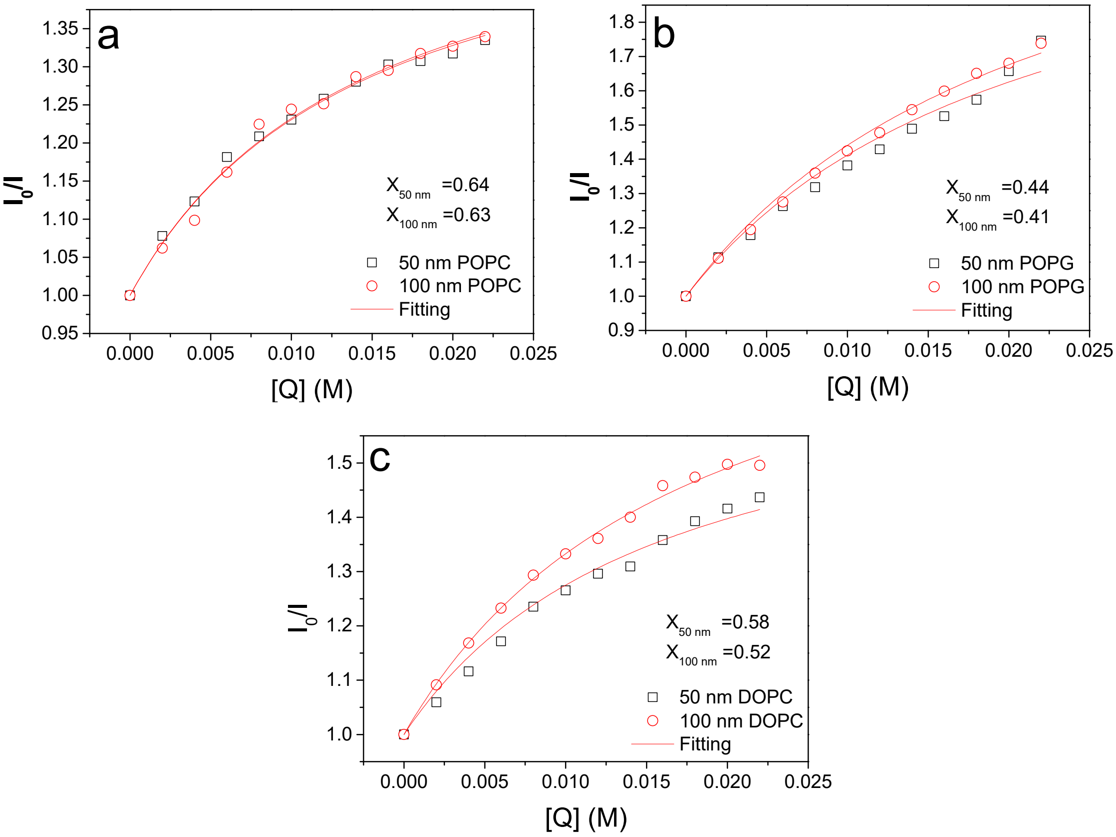

Figure S7. Fluorescence quenching titration data for bR in POPC (**a**), POPG (**b**) and DOPC (**c**) vesicles with different curvatures.

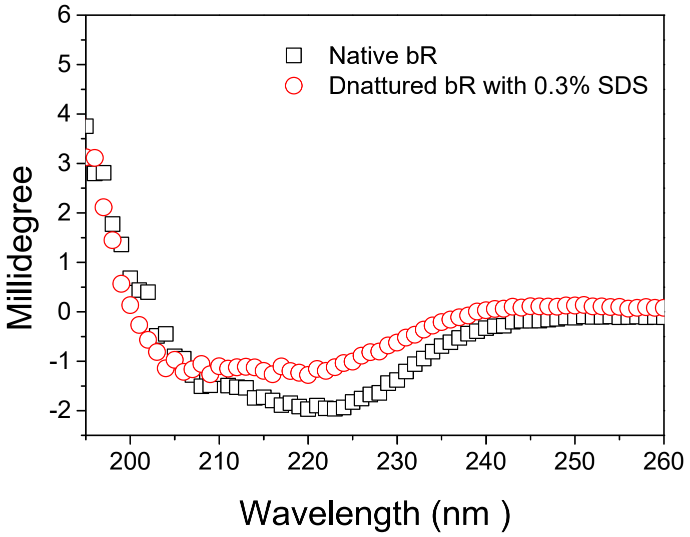

Figure S8. CD spectroscopy of bR proteins with and without SDS, suggesting that bR can be partially denatured by SDS.

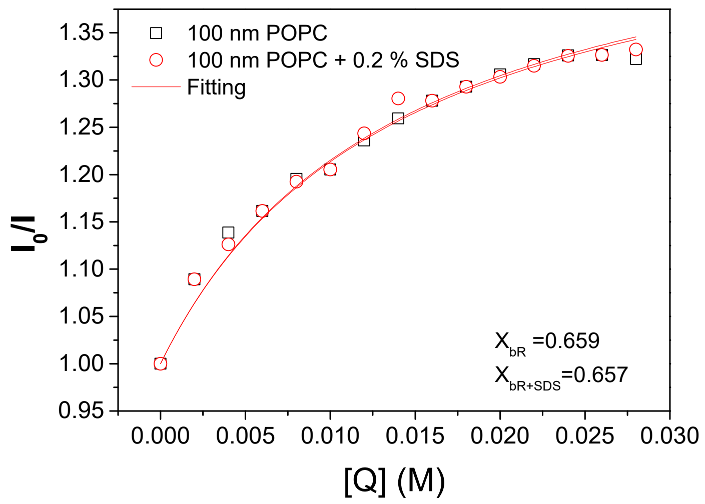

Figure S9. Fluorescence quenching titration data for bR in POPC vesicles with and without SDS.
